# Unsolicited Patient Complaints and Industry Payments for US Physicians

**DOI:** 10.1001/jamanetworkopen.2025.26643

**Published:** 2025-08-05

**Authors:** Yong Hyun Park, Henry John Domenico, Zhuo Tony Su, Bruce J. Trock, Yuezhou Jing, Gerald Hickson, William O. Cooper, Misop Han

**Affiliations:** 1 James Buchanan Brady Urological Institute, Johns Hopkins University School of Medicine, Baltimore, Maryland; 2 Department of Urology, Seoul St Mary’s Hospital, The Catholic University of Korea, Seoul, Republic of Korea; 3 Department of Biostatistics, Vanderbilt University Medical Center, Nashville, Tennessee; 4 Departments of Pediatrics and Health Policy, Center for Patient and Professional Advocacy, Vanderbilt University Medical Center, Nashville, Tennessee

## Abstract

**Importance:**

Both commitment to patient welfare and conflict of interest management are important components of physician professionalism. However, the interplay between them has not been well explored.

**Objective:**

To examine the association between unsolicited patient complaints, measured by Patient Advocacy Reporting System (PARS) Index, and acceptance of general (nonresearch) payments from industry, including significant payments, defined as exceeding $5000 annually.

**Design, Setting, and Participants:**

This retrospective cross-sectional study included physicians at health care sites participating in the PARS program. General payments accepted by physicians from industry were identified from the Open Payments Program database. Data were collected from July 1, 2015, through June 30, 2020, and linked using National Provider Identifier numbers. Data were analyzed from April 5, 2024, to May 12, 2025.

**Exposure:**

The physician’s highest PARS Index score, categorized as 0, 1 to 20, 21 to 50, and 51 or greater.

**Main Outcomes and Measures:**

The outcome of interest was acceptance of general payments, any or exceeding $5000 annually. Ordinal regression was used to assess the association between PARS Index scores and general payments.

**Results:**

This analysis included 71 944 physicians (27 065 [37.6%] female; mean [SD] age in 2015, 45 [12.5] years), with 44 296 (61.6%) practicing in academic settings. The most common specialties were internal medicine (30 043 physicians [41.8%]), general surgery (6819 physicians [9.5%]), and anesthesiology (4461 physicians [6.2%]). Of the included physicians, 49 169 (68.3%) received at least 1 general payment, and 8067 (11.2%) received more than $5000 in a year; 30 979 physicians (43.1%) received unsolicited patient complaints. Median (IQR) PARS Index score was 2 (0-17). A higher PARS Index score was significantly associated with higher general payments accepted per year (eg, PARS Index score ≥51: odds ratio [OR], 1.69; 95% CI, 1.56-1.82; *P* < .001). This association remained significant after adjusting for physician gender, age, region, practice setting, and specialty. Male physicians (OR, 1.90; 95% CI, 1.84-1.97) and physicians practicing at nonacademic settings (OR, 1.15; 95% CI, 1.10-1.19) were also more likely to receive higher general payments per year.

**Conclusions and Relevance:**

In this cross-sectional study of nearly 72 000 physicians across the US, physicians with higher PARS Index scores, indicative of a higher risk of medical malpractice claims, worse patient outcomes, and well-being concerns, were more likely to accept industry payments, particularly in higher amounts. These findings underscore the importance of conflict of interest review and management to support medical professionalism and patient trust.

## Key Points

**Question:**

Do physicians with more unsolicited patient complaints also have a greater likelihood of accepting general (nonresearch) payments from industry?

**Findings:**

In a cross-sectional study of 71 944 physicians, higher Patient Advocacy Reporting System Index scores, reflecting a greater number and severity of unsolicited patient complaints, were significantly associated with an increased likelihood of accepting general payments, particularly in higher amounts.

**Meaning:**

This cross-sectional study found a significant association between unsolicited patient complaints and potential financial conflicts of interest, emphasizing the need for robust conflicts review and management to safeguard patient trust and support physician professionalism.

## Teaser

This cross-sectional study examines associations between unsolicited patient complaints and acceptance of general (nonresearch) payments from industry among US physicians.

## Introduction

Physicians are held to the highest standards of professionalism, guided by a code of ethics and a contract with society. The Charter on Medical Professionalism, developed and adopted by multiple medical societies, identifies the primacy of patient welfare as a fundamental principle of medical professionalism, emphasizing that “market forces, societal pressures, …, must not compromise this principle.”^1^ The charter also underscores the responsibility of physicians to maintain trust by engaging in self and group regulation, including appropriately reviewing and managing conflicts of interest (COIs).^1,2^ Despite the importance of both patient welfare and COI review and management in physician professionalism, it has been challenging to evaluate these principles and understand their interplay.

Unprofessional and disrespectful behaviors modeled by physicians can erode patient-physician relationships, compromise patient welfare, threaten outcomes, and lead to patient complaints.^3,4,5^ The Patient Advocacy Reporting System (PARS), developed at the Vanderbilt Center for Patient and Professional Advocacy, is a reporting and intervention system based on unsolicited patient complaints (UPCs).^6^ Complaints reported to the PARS include those regarding care and treatment, communication, concern for patient and family, accessibility and availability, and payment issues associated with care and treatment.^7^ For each physician, a PARS Index score is calculated using a weighted algorithm emphasizing the recency and severity of UPCs.^7^ The PARS Index score is an established predictor of a physician’s risk of future medical malpractice claims, worse patient outcomes, and well-being concerns independent of clinical volume.^4,8,9,10,11^ Physicians with greater numbers of patient complaints as reflected in a higher PARS Index score have a 3-fold increase in risk of malpractice claims.^4^ Furthermore, the PARS Index also uses a tiered intervention model, with physician peers delivering feedback to physicians with higher risk. This model has proven effective in reducing future patient complaints, reducing medical malpractice claims, and promoting professionalism.^12,13,14^

An effective approach to review and management of COIs is another critical component of physician professionalism. In 2014, the Centers for Medicare & Medicaid Services established the Open Payments Program (OPP), a publicly available, annually updated database designed to enhance transparency regarding financial relationships between physicians, hospitals, and the pharmaceutical and medical device industries.^15^ Between 2015 and 2023, the OPP database reported a total of $72.8 billion in payments from these industries to physicians and hospitals. Of this, $22.3 billion were general payments, including nonresearch, noninvestment payments, such as consulting and speaking fees, gifts, honoraria, meals, and travel expenses.^15^ The acceptance of industry payments has the potential to influence clinical decision-making, raising a concern about financial COIs.^16,17,18,19,20^

We hypothesized that a significant association exists between UPCs and acceptance of payments from industry. Receiving financial payments from industry, especially higher amounts, can lead to COIs for physicians, which could compromise their professional judgement and patient care.^18,19,20^ Conversely, physicians who prioritize their own interests above those of the patients may exhibit behaviors leading to patient dissatisfaction, complaints, and adverse outcomes.^21,22,23,24^ In other words, UPCs and the acceptance of industry payments, particularly in more severe cases, may represent different manifestations of the same underlying physician phenotype. Therefore, in this study we sought to investigate the association of the PARS Index, a predictor of risk of medical malpractice claims, worse patient outcomes, and well-being concerns based on UPCs, with physician acceptance of general payments from industry.

## Methods

### Study Design

This retrospective cross-sectional study received exemption status from review and informed consent from the institutional review boards at Johns Hopkins University and Vanderbilt University according to 45 CFR 46.104(d)(4)(i). This study follows the Strengthening the Reporting of Observational Studies in Epidemiology (STROBE) reporting guideline.

### Data Sources

We collected all data over an observation period of July 1, 2015, through June 30, 2020. We included physicians who practiced at any of the PARS-participating institutions who had at least 1 recorded PARS Index score during the observation period. The PARS Index score was calculated based on a rolling 4 years of UPCs, using an algorithm assigning more weights to more recent and severe complaints.^7^ For early-career physicians for whom a 4-year rolling score was not yet available, we used the PARS Year 1 Index score, calculated based on a rolling 1 year of UPCs also weighted for recency and severity. For each included physician, we used the OPP data to identify acceptance of general (nonresearch) payments from industry during the observation period.^25^ We aggregated general payments accepted by each physician during each academic year, defined as July 1 through June 30, within the 5-year observation period, and total payments accepted over the observation period.

The OPP and PARS datasets were linked using the National Provider Identifier numbers, physician names, and specialty information. Physicians’ birth years were obtained from the American Medical Association Physician Professional Data.^26^ This linkage was conducted by an independent third party not involved in the research process. All analyses were conducted on deidentified datasets to ensure that data could not be linked back to individual physicians.

### Statistical Analysis

We performed all statistical analyses using R software version 4.4.0 (R Project for Statistical Computing) from April 5, 2024, to May 12, 2025. All tests were 2-sided, with statistical significance set at *P* < .05. We used the Pearson χ^2^ test to assess the unadjusted association between the highest PARS Index score observed for a given physician and the highest general payment amount accepted by the physician during a single academic year within the observation period. We categorized the PARS Index scores to reflect low (0), moderate (1-20), high (21-50), and very high (≥51) risk of malpractice claims and surgical complications, following groupings used in previous studies.^4,8^ We categorized general payment accepted by a physician per year into the following groups: $0, $1 to $4999, and $5000 or more. The cutoff of $5000 or more was chosen to reflect the current threshold set by the US Department of Health and Human Services, which considers aggregated payments exceeding $5000 a year to represent a significant financial COI.^27^

In a sensitivity analysis, we used the Kruskal-Wallis test to evaluate the unadjusted association between the highest PARS Index score (≥51) and the highest single-year general payment as a continuous variable. Additionally, we used the Pearson χ^2^ test to assess the association between receipt of any UPC during the observation period and highest single-year general payment category.

We created a multivariable logistic ordinal regression model to evaluate the association between the highest PARS Index score and highest single-year general payment. This model additionally adjusted for physician characteristics, including gender, birth year, practice region (Midwest, Northeast, South, and West), practice setting (academic vs nonacademic), and specialty. Based on prior research showing physicians in surgical subspecialties were more likely to receive general payments and in higher amounts,^28^ we included surgical subspecialties in our specialty categories. We did not adjust the PARS Index score by a physician’s patient volume, based on previous research establishing the PARS Index as a predictor of a physician’s risk of future medical malpractice claims, worse patient outcomes, and well-being concerns independent of clinical volume.^4,8,9,10,11^ For missing values in numerical variables (physician age), median value imputation was used. Missing or unknown values in categorical variables were treated as a separate category (physician gender) or grouped with the existing other category (physician specialty).

## Results

We identified a total of 75 782 physicians practicing at PARS-affiliated institutions during the observation period of July 1, 2015, through June 30, 2020, representing approximately 5% of practicing US physicians; after excluding 3838 physicians (5.1%) with missing PARS Index scores, the final cohort included 71 944 physicians (27 065 [37.6%] female; mean [SD] age in 2015, 45 [12.5] years), with 44 296 (61.6%) practicing in academic settings. The most common specialty was internal medicine (30 043 physicians [41.8%]), followed by general surgery (6819 physicians [9.5%]) and anesthesiology (4461 physicians [6.2%]) (Table 1).

**Table 1.** Demographic Characteristics, UPCs, PARS Index Scores, and Acceptance of General Payments From Industry for a Cohort of US Physicians

Characteristic	Physicians, No. (%)
Gender	
Female	27 065 (37.6)
Male	42 339 (58.9)
Unknown	2540 (3.5)
Age in 2015, y^a^	
Median (IQR)	43 (35-54)
Mean (SD)	45 (12.5)
Practice region	
Midwest	25 013 (34.8)
Northeast	7316 (10.2)
South	17 360 (24.1)
West	22 255 (30.9)
Practice setting	
Academic	44 296 (61.6)
Nonacademic	27 648 (38.4)
Specialty^b^	
Internal medicine	30 043 (41.8)
General surgery	6819 (9.5)
Anesthesiology	4461 (6.2)
Urology	4377 (6.1)
Radiology	4131 (5.7)
Emergency medicine	3615 (5.0)
Orthopedic surgery	3149 (4.4)
Head and neck surgery	2179 (3.0)
Cardiothoracic surgery	618 (0.9)
Plastic surgery	564 (0.8)
Vascular surgery	184 (0.3)
Transplant surgery	79 (0.1)
Other	11 716 (16.3)
Receipt of any UPC^c^	30 979 (43.1)
Total UPCs per physician, No.^c^	
Median (IQR)	0 (0-4)
Mean (SD)	3.4 (6.6)
Highest PARS Index score^c^	
Median (IQR)	2 (0-17)
Mean (SD)	11.6 (18.6)
Highest PARS Index score^c^	
0	35 417 (49.2)
1-20	21 189 (29.5)
21-50	12 251 (17.0)
≥51	3087 (4.3)
Acceptance of any general payment from industry^c^	49 169 (68.3)
Total general payment per physician, $^c^	
Median (IQR)	154 (0-1966)
Mean (SD)	16 877 (201 854)
Highest single-year general payment per physician, $^c^	
Median (IQR)	112 (0-956)
Mean (SD)	6346 (80 697)
Highest single-year general payment per physician^c^	
$0	22 775 (31.7)
$1-$4999	41 102 (57.1)
≥$5000	8067 (11.2)

Abbreviations: PARS, Patient Advocacy Reporting System; UPC, unsolicited patient complaint.

^a^ Age missing or unknown for 17 415 physicians (24.2%).

^b^ Specialty unknown for 9 physicians (<0.1%). Other specialties included neurology or neurosurgery, psychiatry, ophthalmology, pathology, radiology or radiation oncology, and physical medicine and rehabilitation.

^c^ Observation period was July 1, 2015, through June 30, 2020.

During the observation period, 30 979 physicians (43.1%) received at least 1 UPC, with a mean (SD) of 3.4 (6.6) and median (IQR) of 0 (0-4) total complaints per physician. For 1302 physicians (1.8%), a PARS Index score was not available, and a PARS Year 1 Index score was used instead. Physicians were classified based on their highest observed PARS Index score as follows: 35 417 (49.2%) had a maximum score of 0, 21 189 (29.5%) had a score of 1 to 20, 12 251 (17.0%) had a score of 21 to 50, and 3087 (4.3%) had a score above 50. Furthermore, 49 169 physicians (63.8%) accepted at least 1 general payment during the observation period, with a median (IQR) total general payment amount of $154 ($0-$1966) and the mean (SD) total payment amount was $16 877 ($201 854); 8067 physicians (11.2%) received more than $5000 within a year (Table 1).

Table 2 presents the distribution of physicians in terms of receipt of any UPCs and highest observed PARS Index scores, stratified by the highest single-year general payment accepted. The proportion of physicians with at least 1 UPC during the observation period significantly increased with increasing general payment acceptance: 8284 physicians (36.4%) receiving $0, 18 314 physicians (44.6%) receiving $1 to $4999, and 4381 physicians (54.3%) receiving more than $5000 in a year (*P* < .001) had at least 1 UPC. There was a statistically significant positive association between the highest observed PARS Index score and the highest single-year general payment accepted (Table 2). For example, among physicians in the highest PARS Index category, 3.1% of those accepting $0 in general payments fell into this group, compared with 4.5% of those accepting $1 to $4999 and 6.9% of those accepting $5000 or more (*P* < .001). Similarly, the median highest observed PARS Index increased across payment categories: 0 for physicians receiving $0, 4 for those receiving $1 to $4,999, and 8 for those receiving $5000 or more (*P* < .001).

**Table 2.** UPCs and PARS Index Scores for a Cohort of US Physicians

Variable	Physicians by highest single-year general payment, No. (%)^a^			*P* value
	$0 (n = 22 775)	$1-$4999 (n = 41 102)	≥$5000 (n = 8067)	
Receipt of any UPC	8284 (36.4)	18 314 (44.6)	4381 (54.3)	<.001^b^
Highest PARS Index score				
0	12 465 (54.7)	19 856 (48.3)	3096 (38.4)	<.001^b^
1-20	6495 (28.5)	12 209 (29.7)	2485 (30.8)	
21-50	3120 (13.7)	7202 (17.5)	1929 (23.9)	
≥51	695 (3.1)	1835 (4.5)	557 (6.9)	
Highest PARS Index score				
Median (IQR)	0 (0-12)	4 (0-18)	8 (0-26)	<.001^c^
Mean (SD)	9.3 (16.4)	11.9 (18.8)	16.3 (21.8)	

Abbreviations: PARS, Patient Advocacy Reporting System; UPC, unsolicited patient complaint.

^a^ Observation period was July 1, 2015, through June 30, 2020.

^b^ *P* value derived using Pearson χ^2^ test.

^c^ *P* value derived using Kruskal-Wallis test.

In the multivariable logistic ordinal regression model, the positive association between the highest observed PARS Index score and the highest single-year general payment remained statistically significant after adjustment for physician characteristics (odds ratio [OR], 1.69; 95% CI, 1.56-1.82; *P* < .001) . Additionally, male physicians (odds ratio [OR], 1.90; 95% CI, 1.84-1.97), physicians practicing in nonacademic settings (OR, 1.15; 95% CI, 1.10-1.19), and older physicians (OR per 10-year increase in age, 1.02; 95% CI, 1.00-1.03) were significantly more likely to accept higher general payments per year. Physicians practicing in the Midwest were significantly more likely to accept higher payments than those practicing in the other regions (Table 3). Compared with internal medicine physicians, physicians in surgical subspecialties were more likely to accept higher payments, whereas general surgeons, anesthesiologists, radiologists, and emergency medicine physicians were less likely to accept payments (Table 3).

**Table 3.** Multivariable Logistic Ordinal Regression Model for Acceptance of General Payment From Industry

Physician characteristic	OR of receiving ≥$5000 in a year (95% CI)	*P* value
Highest observed PARS Index score		
0	1 [Reference]	NA
1-20	1.20 (1.16-1.24)	<.001
21-50	1.49 (1.43-1.55)	<.001
≥51	1.69 (1.56-1.82)	<.001
Gender		
Female	1 [Reference]	NA
Male	1.90 (1.84-1.97)	<.001
Unknown	0.14 (0.13-0.15)	<.001
Age as of 2015, per 10-y increase	1.02 (1.002-1.03)	.03
Practice region		
Midwest	1 [Reference]	NA
Northeast	0.89 (0.84-0.94)	<.001
South	0.95 (0.91-0.99)	.04
West	0.92 (0.88-0.96)	<.001
Practice setting		
Academic	1 [Reference]	NA
Nonacademic	1.15 (1.10-1.19)	<.001
Physician specialty		
Internal medicine	1 [Reference]	NA
General surgery	0.85 (0.80-0.90)	<.001
Anesthesiology	0.62 (0.58-0.66)	<.001
Urology	1.41 (1.32-1.50)	<.001
Radiology	0.08 (0.08-0.09)	<.001
Emergency medicine	0.27 (0.25-0.29)	<.001
Orthopedic surgery (nonspine)	1.96 (1.68-2.27)	<.001
Orthopedic surgery (spine)	3.72 (3.42-4.04)	<.001
Head and neck surgery	1.33 (1.22-1.46)	<.001
Cardiothoracic surgery	4.01 (3.43-4.70)	<.001
Plastic surgery	1.90 (1.61-2.25)	<.001
Vascular surgery	3.04 (2.29-4.04)	<.001
Transplant surgery	1.70 (1.08-2.68)	.02
Other or unknown specialty^a^	0.86 (0.82-0.90)	<.001

Abbreviations: NA, not applicable; OR, odds ratio; PARS, Patient Advocacy Reporting System.

^a^ Other specialties included neurology or neurosurgery, psychiatry, ophthalmology, pathology, radiology or radiation oncology, and physical medicine and rehabilitation.

## Discussion

In this cross-sectional study of nearly 72 000 physicians across the US, we identified a statistically significant association of higher PARS Index scores, based on patient complaints, with acceptance of higher general payments from industry. This association remained significant after adjusting for demographic and practice-related factors. To our knowledge, this is the first study to demonstrate a positive association between patient complaints and general payments from industry for physicians. Our study builds on previous studies that have shown that payments from pharmaceutical or medical device companies may influence physicians’ treatment decision-making and especially prescribing behaviors,^16,18,19,20,29^ which may lead to adverse outcomes and patient dissatisfactions.^22,23,24^

Another notable finding was that male physicians and those practicing in nonacademic settings were more likely to accept larger general payments. Gender disparity in industry payments have been demonstrated previously, with one explanation being that female physicians may have fewer opportunities for industry engagement due to time constraints or being offered fewer opportunities to receive payments by industry.^30^ Alternatively, female physicians may exhibit personal preferences that align with avoiding financial COIs with industries.^30^ Similarly, physicians in academic practice settings accepted fewer general payments, likely due to stricter institutional COI policies at academic medical centers.^31^

Our study underscores the urgency for health care institutions to address the dual challenges of patient complaints and financial COI management. Efforts to strengthen physician professionalism should include enhanced transparency and targeted interventions, such as professionalism training, to mitigate potential conflicts and strengthen trust between physicians and patients.^12,32,33^ Initiatives such as the Medical Professionalism Project and the Charter on Medical Professionalism offer valuable frameworks for fostering this culture.^1^

### Limitations

Our study has several limitations. First, the observational nature of this study precludes causal inference; however, such an approach is well-suited for examining complex associations. The consistency of the observed associations aligns with prior research that has highlighted associations between financial COIs and physician behaviors.^18,19,20,34^ Second, the use of secondary datasets introduces the potential for misclassification bias, particularly if industry payments were underreported. However, reporting is expected to be robust, given the significant financial penalties for misrepresentation.^35,36^ Additionally, our analysis is limited by assessing only the total general payments accepted by physicians, without stratifying by individual types of payments (eg, travel expenses, honoraria, speaking fees, gifts). Furthermore, our analysis is likely affected by residual confounding. For example, more granular stratifications can be applied to physician demographic and practice factors, such as physician specialty, where various subspecialties within internal medicine are grouped together. Additionally, unmeasured confounders, such as differences in institutional policies or regional practice patterns, may influence the observed associations.

## Conclusions

In this cross-sectional study of nearly 72 000 physicians across the US, physicians with higher PARS Index scores, reflective of a greater number and severity of UPCs, were more likely to receive general payments from industry, especially in higher amounts, defined as exceeding $5000 annually. This finding highlights the complex interplay between patient complaints and financial COIs among physicians. The observed positive association between higher PARS Index scores and the acceptance of general payments underscores potential vulnerabilities in physician professionalism. These findings emphasize the need for multifaceted strategies to address lapses in professionalism, safeguard the integrity of clinical decision-making, and strengthen patient trust. Future research should focus on evaluating the effectiveness of targeted interventions, such as peer-guided feedback to physicians who are outliers and COI review and management, to mitigate these challenges and promote a culture of accountability and transparency in health care.

## Notes

Supplement.
Data Sharing Statement
