# Supplementary material for: Unsolicited Patient Complaints and Industry Payments for US Physicians
Source: JAMA Netw Open. 2025 Aug 5;8(8):e2526643. doi: 10.1001/jamanetworkopen.2025.26643 (PMC12326278; doi:10.1001/jamanetworkopen.2025.26643)
Supplement: Supplement. — Data Sharing Statement [file jamanetwopen-e2526643-s001.pdf]

Data Sharing Statement

Park. Unsolicited Patient Complaints and Industry Payments for US Physicians. *JAMA Netw Open*. Published August 05, 2025. doi:10.1001/jamanetworkopen.2025.26643

Data
Data available: No

Additional Information
Explanation for why data not available: There are multiple contracts between healthcare institutions and PARS/Vanderbilt University that prohibit the disclosure of individual physician information.
